# Elevated ratio of MMP2/MMP9 activity is associated with poor response to chemotherapy in osteosarcoma

**DOI:** 10.1186/s12885-016-2266-5

**Published:** 2016-03-15

**Authors:** Pierre Kunz, Heiner Sähr, Burkhard Lehner, Christian Fischer, Elisabeth Seebach, Jörg Fellenberg

**Affiliations:** Clinic for Orthopedics and Trauma Surgery/Spinal Cord Injury Center; Center for Orthopedics, Trauma Surgery and Spinal Cord Injury, University Hospital Heidelberg, Heidelberg, Germany

**Keywords:** Osteosarcoma, Matrix metalloproteinases, Tissue inhibitor of matrix metalloproteinases, Zymography, Chemotherapy, Prognosis

## Abstract

**Background:**

Matrix metalloproteinases (MMPs) are crucially involved in the regulation of multiple stages of cancer progression. Elevated MMP levels have been associated with the development of metastases and poor prognosis in several types of cancer. However, the role of MMPs in osteosarcoma and their prognostic value is still unclear. Available data are conflicting, most likely due to different technical approaches. We hypothesized that in contrast to total mRNA or protein levels frequently analyzed in previous studies the enzymatic activities of MMPs and their inhibitors the tissue inhibitors of matrix metalloproteinases (TIMPs) are closer related to their biological functions. We therefore aimed to evaluate the reliability of different zymography techniques for the quantification of MMP and TIMP activities in osteosarcoma biopsies in order to investigate their distribution, possible regulation and prognostic value.

**Methods:**

All analyses were done using cryo-conserved osteosarcoma pretreatment biopsies (*n* = 18). Gene and protein expression of MMPs and TIMPs were analyzed by RT-qPCR and western blot analysis, respectively. Overall MMP activity was analyzed by in situ zymography, individual MMP activities were analyzed by gelatin zymography. Reverse zymography was used to detect and quantify TIMP activities.

**Results:**

Strong overall MMP activities could be detected in osteosarcoma pretreatment biopsies with MMP2 and MMP9 as predominant active MMPs. In contrast to total RNA or protein expression MMP2 and MMP9 activities showed significant quantitative differences between good and poor responders. While MMP9 activity was high in the good responder group and significantly decreased in the poor responder group, MMP2 activity showed a reverse distribution. Likewise, significant differences were detected concerning the activity of TIMPs resulting in a negative correlation of TIMP1 activity with MMP2 activity (*p* = 0.044) and negative correlations of TIMP2 and TIMP3 with MMP9 activity (*p* = 0.007 and *p* = 0.006).

**Conclusion:**

In contrast to mRNA or protein levels MMP and TIMP activities showed significant differences between the analyzed good and poor responder groups. A shift from MMP9 to predominant MMP2 activity is associated with poor response to chemotherapy suggesting that the ratio of MMP2/MMP9 activity might be a valuable and easily accessible marker to predict the response to chemotherapy in osteosarcoma.

## Background

Osteosarcoma is the most common primary tumor of the bone, typically affecting the long tubular bones of children and adolescents. The introduction of effective neoadjuvant and adjuvant chemotherapy significantly improved outcome with long-term relapse free survival rates ranging from 55 to 75 % over the last several decades [1, 2]. However, the remainder of patients will relapse, most often with pulmonary metastases and poor response to chemotherapy [3]. Therefore, stratification of patients at time of diagnosis into low and high risk groups is highly warranted to improve outcome of high-risk patients and minimize toxicity of therapy for low-risk patients by means of a risk-adapted therapy. Histologic response of the tumor to chemotherapy is still the most reliable prognostic factor, but cannot be assessed at the time of diagnosis.

Matrix metalloproteinases (MMPs) are proteolytic enzymes that play a major role in extracellular matrix (ECM) remodeling but have also been shown to be involved in the regulation of multiple stages of cancer progression including cell growth, differentiation, apoptosis, migration, invasion and immune surveillance [4, 5]. Elevated MMP levels have been shown to be associated with metastasis and poor prognosis in several types of cancer [6, 7]. MMPs further play an important role in the regulation of angiogenesis. Besides the degradation of the ECM that promotes the detachment and migration of endothelial cells, MMPs contribute to the release of proangiogenic factors like bFGF, VEGF and TGF-ß from the ECM [8].

Regulation of MMP activity is a complex process that occurs at multiple levels including gene expression, proteolytic activation of the latent pro-forms of the enzymes and interaction with their physiological inhibitors, the tissue inhibitors of matrix metalloproteinases (TIMPs) [9]. The TIMP family consists of four members (TIMP-1, −2, −3, −4) that are, besides inhibition of MMP activity, involved in several cellular processes including inhibition of apoptosis and activation of cell survival pathways [10]. The role of MMPs and TIMPs in osteosarcoma and their prognostic value is still unclear and available data are conflicting. Most studies so far focused on the analysis of gene and protein expression while data on enzyme activity are very limited. In addition, the prognostic value of MMPs has been evaluated mainly concerning overall and event-free survival. Associations of MMPs with response to chemotherapy have not been studied yet.

Therefore, the aim of our study was to evaluate the reliability of different zymography techniques for the quantification of MMP and TIMP activities in order to investigate their distribution in osteosarcoma tissue and to reveal possible prognostic properties.

## Methods

### Patients and treatment

All patients included in our study were diagnosed for osteosarcoma by open tumor biopsy between 2005 and 2012 and received equal neoadjuvant chemotherapy according to the standard recommendations within the EURAMOS-1 (European and American osteosarcoma study group) treatment study. The neoadjuvant treatment is based on high dose Methotrexate, Doxorubicin and Cisplatin. Following tumor resection patients are stratified for further adjuvant chemotherapy according to their response to neoadjuvant chemotherapy.

Our study design, investigating enzyme activity of MMPs and TIMPs, demanded fresh frozen tumor tissue of adequate quality. Out of 37 treatment naive fresh frozen osteosarcoma samples collected between 2005 and 2012 in our bone tumor tissue bank, sufficient clinical data were available for 31 cases. In four cases, the quantity of the preserved tumor tissue was not sufficient for further investigations, and nine fresh frozen tumor samples demonstrated significant signs of degradation of RNA and proteins. Therefore 18 samples remained available for further investigations. Response to preoperative chemotherapy was assessed histologically by the pathologist according to the six-grade scale of Salzer-Kuntschik [11] where grade 1 denotes no viable tumor cells; grade 2; solitary viable cells or one islet of less than 0.5 cm; grade 3; less than 10 %; grade 4; 10 % to 50 %; grade 5; more than 50 % and grade 6; no effect of chemotherapy.

In this classification a good response is defined as less than 10 % viable tumor cells corresponding to response grades 1 through 3. Reflecting approximately the distribution of poor and good responders observed in larger studies [12], nine good and nine poor responders were identified within our included patients.

### RNA extraction

For quantitative RT-PCR analyses total RNA was extracted from cryoconserved tumor samples. Frozen tissue was pulverized in a Micro-Dismembrator-S (Braun, Melsungen, Germany) and resuspended in RNA lysis buffer included in the mirVana RNA isolation kit (Invitrogen, Darmstadt, Germany). All further steps were performed according to manufacturer protocol. RNA purity and concentration were determined with a NanoDrop ND-100 spectrometer (PeqLAb, Erlangen, Germany).

### cDNA synthesis and quantitative RT-PCR

First strand complementary DNA (cDNA) was synthesized from 1 μg of total RNA using 1 μl Omniscript (Qiagen, Hilden, Germany), 10 μM oligo-dT primer, 5 mM dNTPs and 10U RNaseOut (Invitrogen, Karlsruhe, Germany) for 2 h at 37 °C in a total volume of 20 μl. Quantitative real-time PCR was performed in the real-time thermal cycler Mx3005p (Agilent Technologies, Waldbronn, Germany) in a total volume of 20 μl using Absolute QPCR SYBR Green mix (Thermo scientific, Dreieich, Germany) and 1 μl of cDNA as template. Samples were heated to 95 °C for 15 min followed by 40 cycles of denaturation at 95 °C for 15 s, annealing at 58 °C for 20 s and extension at 72 °C for 30 s. After the last cycle, a melting curve analysis was performed to verify the specificity of the amplified PCR products. Calculated gene expression was normalized on the basis of the expression of RPL19 (ribosomal protein L19) in the corresponding sample. The following primers were used: TIMP1-F: 5′-GGGCTTCACCAAGACCTACA-3′, TIMP1-R: 5′-TGCAGGGGATGGATAAACAG-3′, TIMP2-F: 5′-GAAGAGCCTGAACCACAGGT-3′, TIMP2-R: 5′-CGGGGAGGAGATGTAGCAC-3′, TIMP3-F: 5′-GTGCAACTTCGTGGAGAGGT-3′, TIMP3-R: 5′-AGCAGGACTTGATCTTGCAGT-3′, MMP2-F: 5′-ATAACCTGGATGCCGTCGT-3′, MMP2-R: 5′-AGGCACCCTTGAAGAAGTAGC-3′, MMP9-F: 5′-GAACCAATCTCACCGACAGG-3′, MMP9-R: 5′-GCCACCCGAGTGTAACCATA-3′,, RPL19-F: 5′-GTGGCAAGAAGAAGGTCTGG-3′, RPL19-R: 5′-GCCCATCTTTGATGAGCTTC-3′

### Protein extraction

For isolation of total proteins from osteosarcoma biopsies, cryo-conserved tissue was pulverized in a Micro-Dismembrator-S (Braun) and resuspended in RIPA lysis buffer (Santa Cruz, Heidelberg, Germany) without the addition of protease inhibitors. Lysates were further homogenized two times by sonication for 30 s on ice. Lysates were incubated at 4 °C for 1 h and centrifuged at 12000 g for 10 min to remove cellular debris. Supernatants were recovered and protein concentrations were determined by BCA assay (Fisher Scientific, Schwerte, Germany).

### Western blot

For the detection of TIMP and MMP protein expression 15 μg of total protein was separated on a 10 % polyacrylamide gel and transferred to Immobilon-P membranes (Millipore, Schwalbach, Germany). After blocking in PBS supplemented with 5 % skim milk (Sigma-Aldrich) and 0.05 % Tween 20 (Sigma-Aldrich) membranes were incubated overnight at 4 °C with one of the following primary antibodies at the indicated dilutions: MMP2 (1:500) (Abcam, Cambridge, United Kingdom), MMP9 (1:500) (Abcam) TIMP1 (1:250) (Abcam), TIMP2 (1:250) (Abcam), TIMP3 (1:250) (Abcam). After washing three times in PBS containing 0.1 % Tween 20 membranes were incubated for 1 h at room temperature with 5000-fold diluted peroxidase conjugated goat anti-mouse IgG (Santa Cruz). Proteins recognized by the antibody were visualized with luminata forte western blotting substrate (Millipore) according to the manufacturer’s instructions. Signal intensities were quantified by densitometry using Bio-1D software version 15.01 (Vilber Lourmat, Eberhardzell, Germany).

### Gelatin zymography and reverse zymography

For the analysis of MMP activities in osteosarcoma tissue, equal amounts of total proteins (10 μg) were separated without heating of the samples and without addition of ß-mercaptoethanol on a 10 % SDS-polyacrylamid gel including 0.15 % gelatin (Sigma-Aldrich, München, Germany). As positive control culture supernatant from MG63 osteosarcoma cells was used. As negative control RIPA lysis buffer was loaded on the gel. Gels were washed two times in 2.5 % Triton-X 100 for 20 min and incubated for 16 h at 37 °C in developing buffer consisting of 150 mM NaCl, 5 mM CaCl_2_ and 50 mM Tris pH8.0 (Carl-Roth, Karlsruhe, Germany). After staining with 0.5 mg/ml Coomassie Brilliant Blue R-250 (Sigma) in 10 % acetic acid and 25 % methanol for 2 h with gentle agitation, gels were destained for further 2 h in 8 % acetic acid and 4 % methanol and photographed. Signal intensities were quantified by densitometry using Bio-1D software version 15.01 (Vilber Lourmat, Eberhardzell, Germany).

Reverse zymography was performed as described above, except that the polyacrylamide gels were supplemented with culture supernatant from osteosarcoma MG63 cells containing MMP2. The culture supernatant was concentrated 6-fold by Centriplus YM-3 centrifugal filter devices before 500 μl was added to 7.5 ml gel. Incubation, staining and destaining was performed as described for gelatin zymography. As positive control 100 ng recombinant TIMP1 and 100 ng TIMP2 (PeproTech, Hamburg, Germany) were combined and loaded on the gel. As negative control RIPA lysis buffer was used.

### In situ zymography

In situ zymography was performed on 10 μm cryosections of osteosarcoma pretreatment biopsies. Sections were thawed, and equilibrated for 10 min at 20 °C in incubation buffer consisting of 50 mM Tris pH 7.4, 150 mM NaCl and 5 mM CaCl_2_. Sections were then covered with 100 μl of incubation buffer supplemented with (50 μg/ml) FITC conjugated DQ gelatin as substrate (Life Technologies, Darmstadt, Germany). After incubation for 16 h at 37C in a wet chamber sections were washed 3 times for 5 min in TBS buffer and mounted using Roti-Mount FluorCare (Carl-Roth) containing DAPI as nuclear counterstain. For negative controls CaCl_2_ in the incubation buffer was replaced by 10 mM EDTA.

### Statistics

Statistical analysis was done using SPSS 22 software. Statistical comparison of experimental groups was done using a Mann-Whitney-U test. Calculation of correleation coefficients of MMP and TIMP activities was done using a Pearson correlation analysis. Results were defined as significant if *p* < 0.05 and highly significant if *p* < 0.01.

## Results

**Fig. 1:**
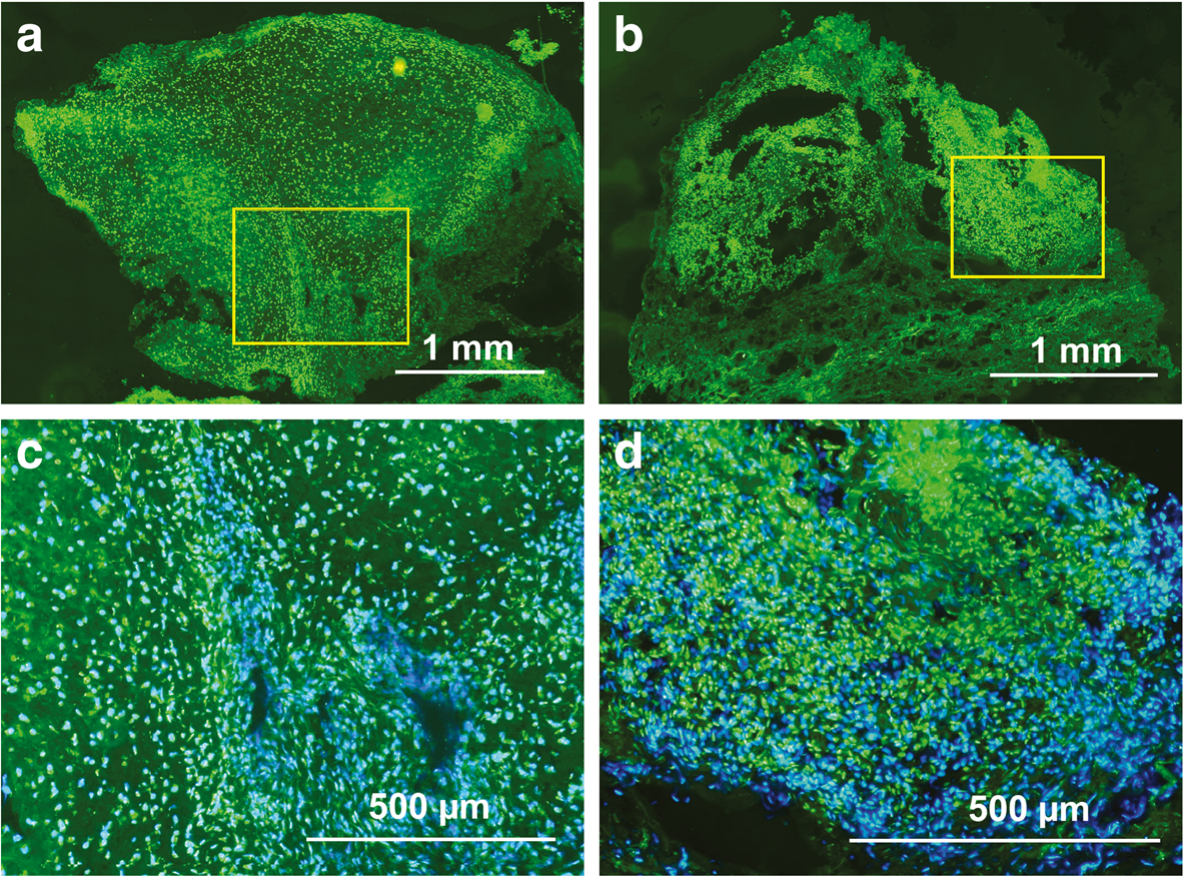
Analysis of total MMP activity in osteosarcoma biopsies by in situ zymography. Green fluorescent areas represent MMP mediated degradation of FITC-labeled substrate. Representative in situ zymographies of cryosections derived from (**a**) good responder and (**b**) poor responder are shown. Yellow frames indicate the area of magnification shown in (**c**) and (**d**). DAPI counterstain of nuclei is shown in blue

**Fig. 2:**
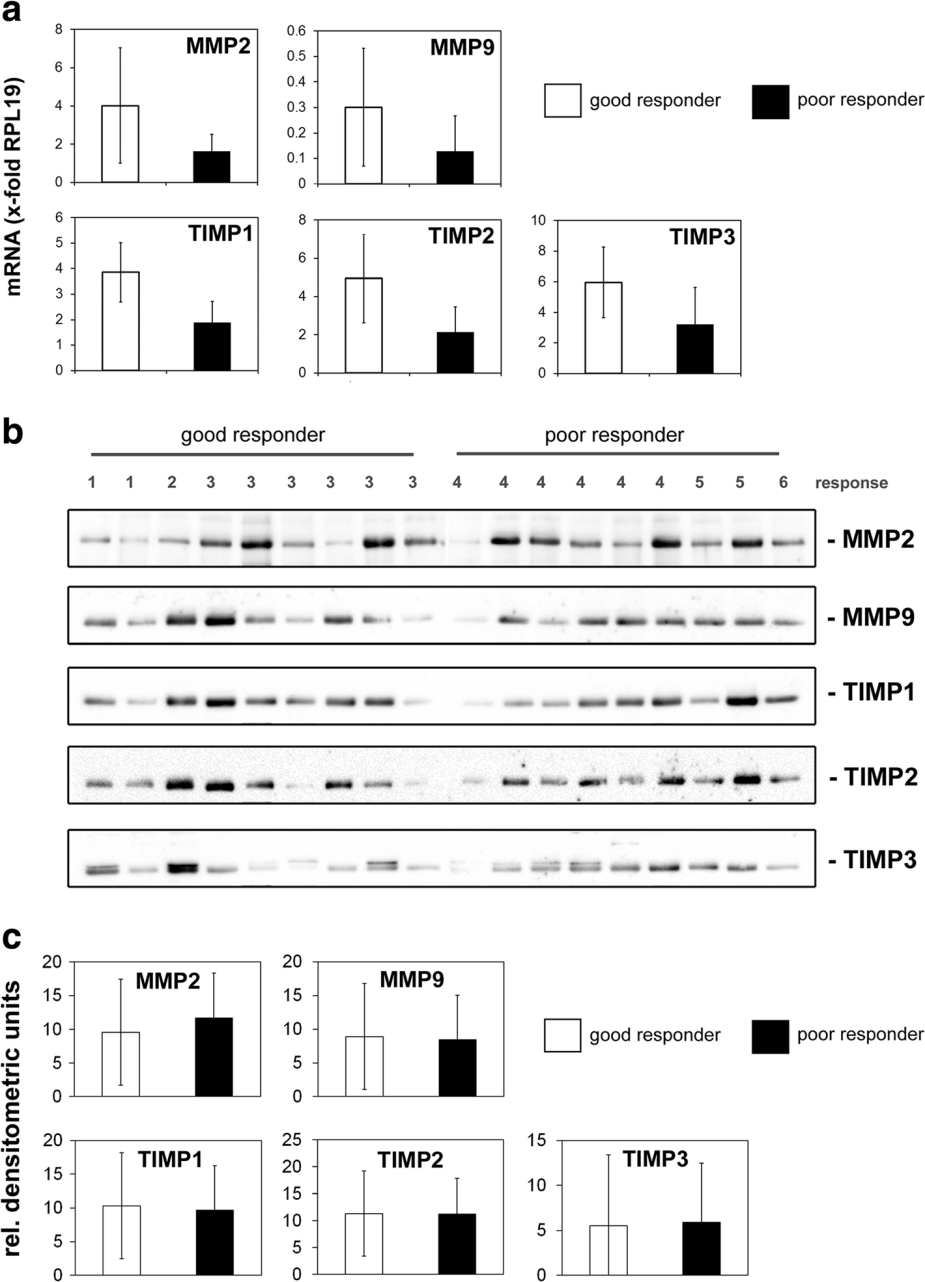
Quantitative analysis of *MMP2, MMP9, TIMP1, TIMP2* and *TIMP3* gene and protein expression. **a** Real-time RT-qPCR analysis of *MMP2, MMP9, TIMP1, TIMP2 and TIMP3* gene expression in osteosarcoma biopsies from good and poor responders (*n* = 9 each). Expression levels were normalized to the expression of *RPL19* (ribosomal protein L19) in the corresponding sample. **b** Western blot analysis of MMP2, MMP9, TIMP1, TIMP2 and TIMP3 protein expression in osteosarcoma biopsies from good and poor responders (*n* = 9 each). Response to preoperative chemotherapy according to the six-grade scale of Salzer-Kuntschik is indicated. **c** Densitometric quantification of the western blots shown in (**b**). The mean values ± SD of the analyzed good and poor responder groups are shown

Total MMP activity was analyzed by in situ zymography on cryosections derived from osteosarcoma pretreatment biopsies. As substrate, a highly quenched FITC labeled gelatin was used that does not produce any fluorescence in the absence of MMP activity. Upon MMP induced proteolytic digestion a bright, green fluorescence is revealed suitable for densitometric quantification of total enzyme activity. Using this technique we observed a strong total MMP activity in all analyzed samples. The majority of MMP activity was located on the cell surface and less activity was found in the ECM. Densitometric quantification of the MMP positive area within the tissue sections did not detect any significant differences between the good (11.4 %) and poor responder group (12.5 %) (Fig. 1). To identify individual differences we next analyzed RNA and protein expression of MMP2, MMP9, TIMP1, TIMP2 and TIMP3 by RT-qPCR and western-blot analysis. Expression of all genes could be detected in the analyzed samples. However, although all genes showed a trend to be downregulated in the poor responder group we could not detect any significant differences between the analyzed groups (Fig. 2a).

Likewise, protein expression could be detected in all samples but densitometric quantification of the protein bands did not detect any significant differences between the analyzed groups (Fig. 2 b + c).

**Fig. 3:**
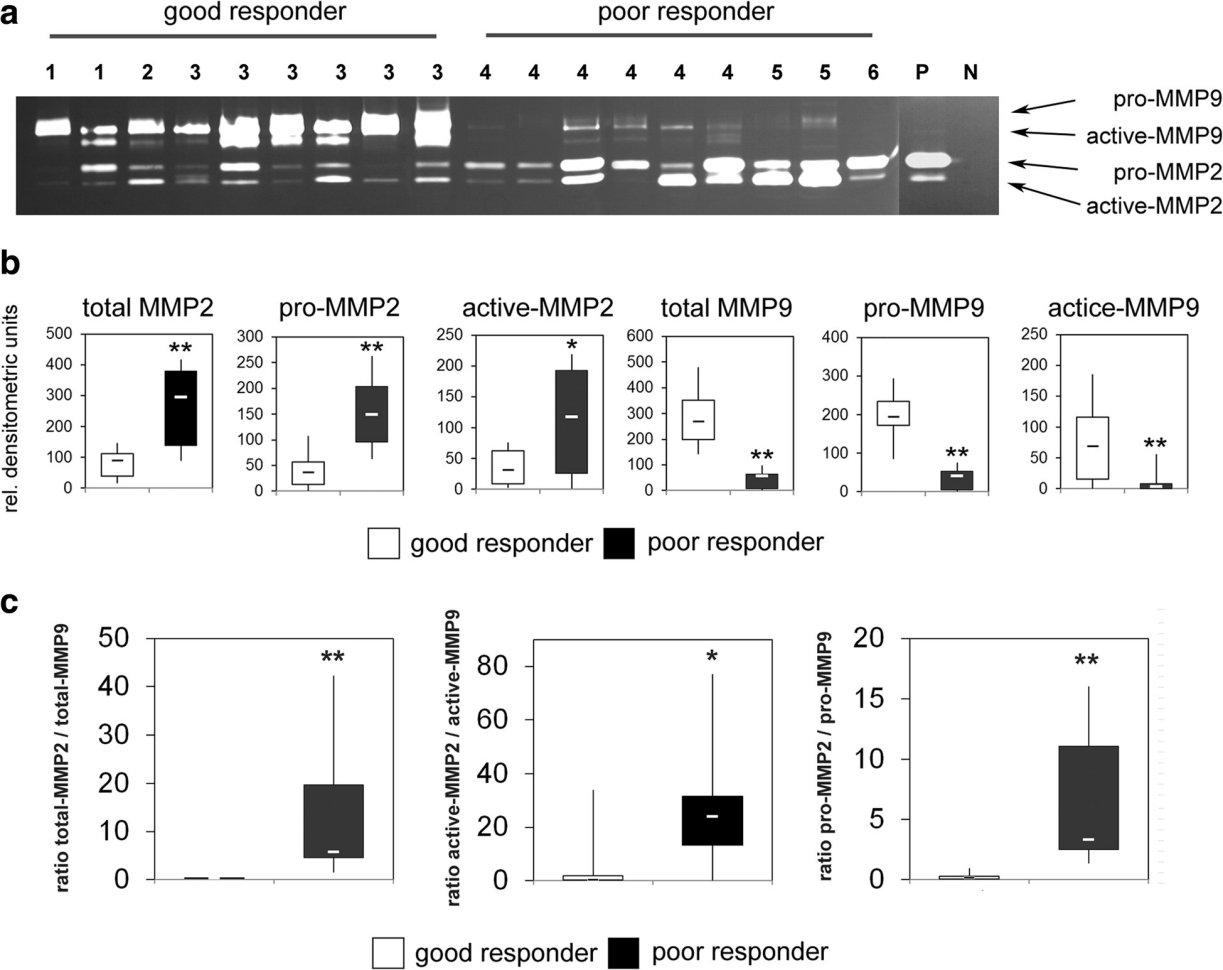
**a** Gelatine zymography of total protein lysates from osteosarcoma pretreatment biopsies. Latent pro- and active forms of MMP2 and MMP9 were detected and are marked by arrows. Response to preoperative chemotherapy according to the six-grade scale of Salzer-Kuntschik is indicated. (*P =* positive control, *N =* negative control) (**b**) Densitometric quantification of pro- and active forms as well as the total amount of MMP2 and MMP9 in the good and poor responder group. The lower boundary of the box indicates the 25^th^ percentile, and the upper boundary the 75^th^ percentile. The whiskers indicate the highest and lowest values. Medians are indicated. *P*-values were determined by Mann-Whitney U-test (**p < 0.01, *p < 0.05). **c** Ratios of the active forms, the latent pro-forms and total MMP2/MMP9 in osteosarcoma biopsy samples from good and poor responders. *P*-values were determined by Mann-Whitney U-test (**p < 0.01, *p < 0.05)

**Fig. 4:**
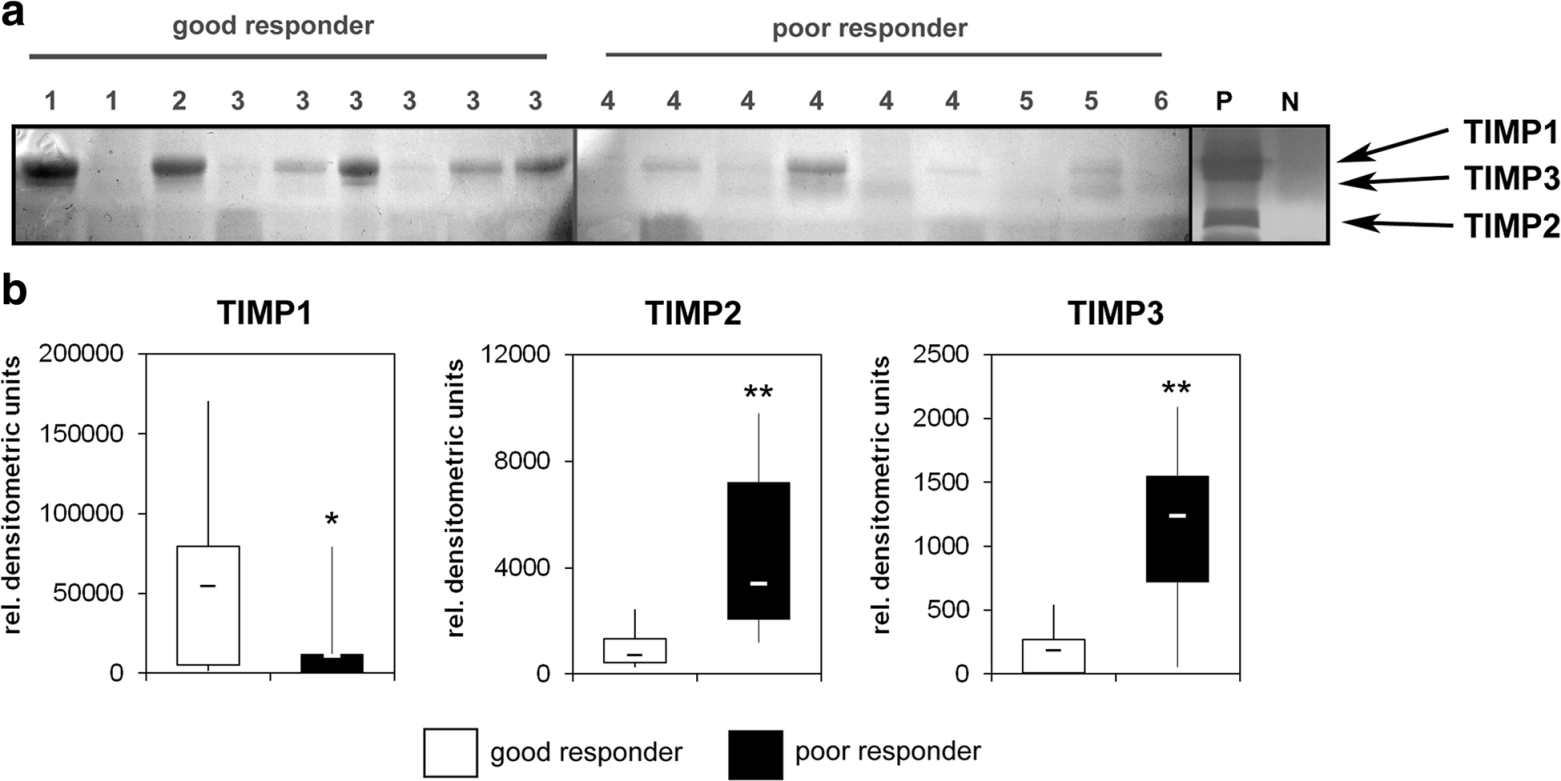
**a** Reverse zymography of total protein lysates from osteosarcoma pretreatment biopsies. Equal amounts of protein were loaded on the acrylamide gel supplemented with MMP2 and MMP9. TIMP mediated inhibition of MMP activity was detected as dark bands. Based on their molecular weight TIMP1, TIMP2 and TIMP3 could be identified and are marked by arrows. (*P =* positive control, *N =* negative control) (**b**) Densitometric quantification of TIMP1, TIMP2 and TIMP3. The lower boundary of the box indicates the 25^th^ percentile, and the upper boundary the 75^th^ percentile. The whiskers indicate the highest and lowest values. Medians are indicated. P-values were determined by Mann-Whitney U-test (**p < 0.01, *p < 0.05)

**Fig. 5:**
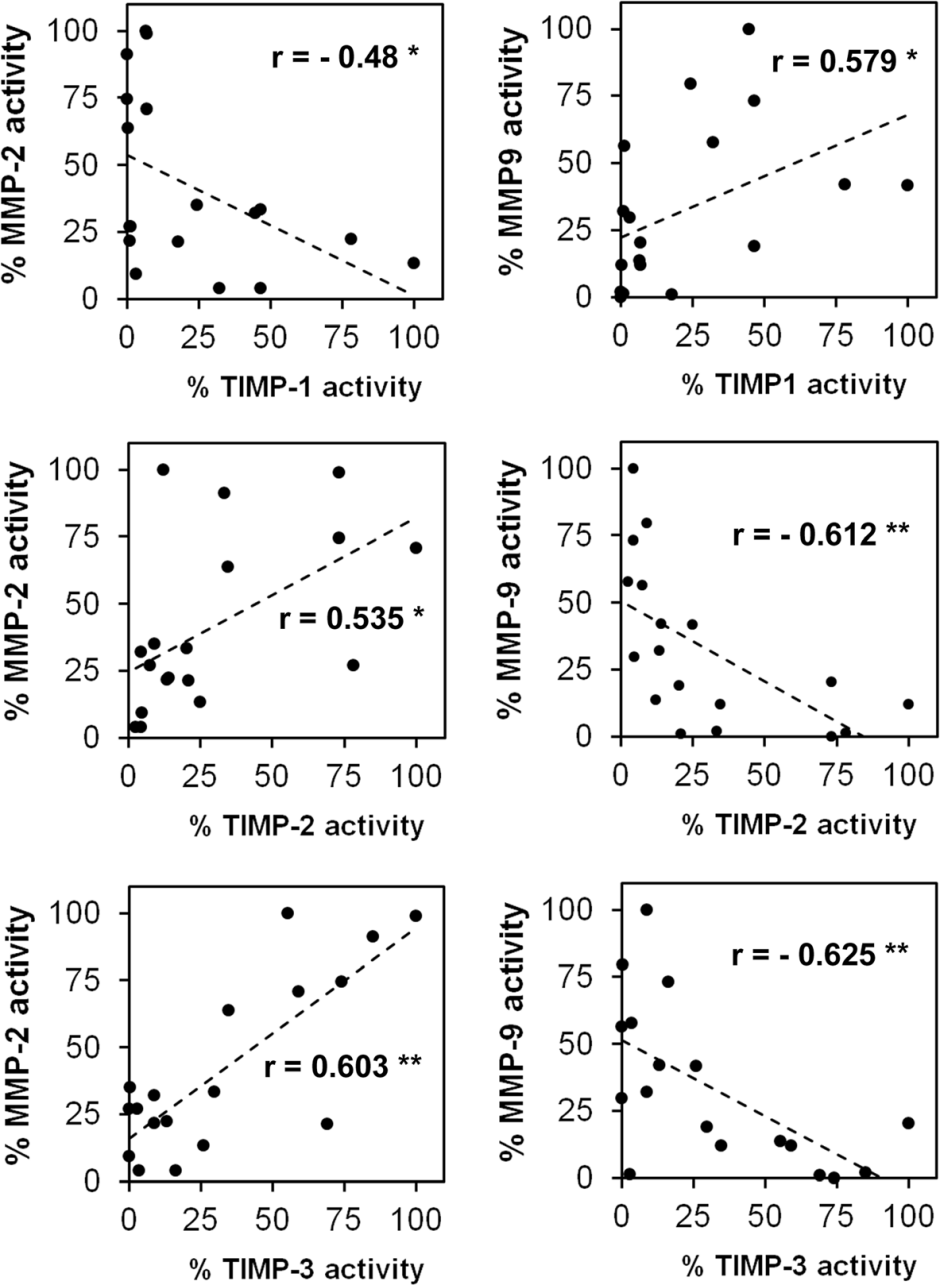
Correlations of MMP and TIMP activities in osteosarcoma biopsies. MMP and TIMP activities were quantified by zymography, plotted against each other and analyzed by Pearson Correlation. Correlation coefficients and p-values are indicated (**p < 0.01, *p < 0.05)

To verify our hypothesis that the analysis of MMP and TIMP activities is more informative concerning clinical parameters than total RNA or protein levels we next analyzed MMPs and TIMPs in protein lysates by zymography. Based on the different size of the proteins we could detect the pro- and active forms of MMP2 and MMP9 in all analyzed samples. The activities of both MMPs showed significant quantitative differences between the good and poor responder group. While MMP9 activity was high in the good responder group and low in the poor responder group, MMP2 activity showed a reverse distribution (Fig. 3a). Quantitative analysis of the detected MMP activities by densitometry confirmed these observations. While the amount of active MMP2 increased 3.2-fold in the poor responder group, the amount of active MMP9 decreased 7.6-fold in this group. Comparable changes were observed for the total activities of MMP2 and MMP9 as well as the latent pro-forms of these enzymes that are activated during zymography by SDS-mediated denaturation and subsequent renaturation of the zymogens (Fig. 3b). As a consequence, the mean ratio of total-, active- and pro-MMP2/MMP9 activity significantly increased 23-fold (*p* = 0.00059), 59-fold (*p* = 0.0378) and 33-fold (*p* = 0.00026), respectively in the poor responder group. (Fig. 3c). Using the calculated medians of the MMP ratios as cutoff value a correct prediction of chemotherapy response would have been possible in 100 % (total-MMPs), 100 % (pro-MMPs) and 89 % (active-MMPs) of cases. To analyze, whether modulated TIMP activities may be responsible for the observed differences in MMP activities we performed reverse zymography with the same samples. In this assay, MMPs derived from culture supernatants of an osteosarcoma cell line were incorporated together with gelatin into the polyacrylamide gel. TIMPs within the analyzed protein lysates inhibited the MMP induced gelatin degradation and could thus be visualized as dark bands after coomassie blue staining [13]. The strongest signals could be detected for TIMP1. Densitometric quantification revealed a 4.9-fold higher mean activity of TIMP1 in the good responder group compared to poor responders. In contrast, detected TIMP2 and TIMP3 activities were markedly lower and showed a reverse distribution. Mean TIMP2 activity was 4.7-fold higher in the poor responder group and mean TIMP3 activity was elevated 6.6-fold compared to the good responder group (Fig. 4a + b). In addition, we could detect significant correlations between TIMP and MMP activities. TIMP1 activity showed a positive correlation to MMP9 activity (*p* = 0.012) and a negative correlation of to MMP2 activity (*p* = 0.044). In contrast TIMP2 and TIMP3 activities were negatively correlated to MMP9 activity (*p* = 0.007 and *p* = 0.006) and showed a positive correlation to MMP2 activity (*p* = 0.022 and *p* = 0.000093) (Fig. 5).

## Discussion

Numerous studies have demonstrated that MMPs play critical roles in tumor growth and progression, the development of metastases, angiogenesis and tumor invasion [14–16] Elevated MMP levels have been associated with poor prognosis in several types of tumors including breast cancer, gastric cancer and osteosarcoma [17–21]. Among the more than 20 known members of the MMP family, especially MMP2 and MMP9 have been extensively studied in human cancers and have been shown to be closely related to the invasive potential and metastasis of different types of tumor cells including osteosarcoma [22, 23]. As a consequence, increased expression of MMP2 and MMP9 have been associated with a more aggressive phenotype and poor prognosis in several solid tumors including breast cancer [24], ovarian carcinoma [25], head and neck squamous cell carcinoma [26] and gastric cancer [20]. However, the role of MMPs in osteosarcoma and their value as prognostic marker is still unclear and available data are conflicting. Most studies focused on the involvement of MMPs in the metastatic process of osteosarcomas based on their capacity to degrade ECM and basement membranes, facilitating cellular motility and invasion. In this context, upregulation of MMP9 but not MMP2 has been shown to contribute to increased metastatic ability of osteosarcoma cells [27]. Furthermore, MMP9 activity and gene expression has been reported to correlate with the metastatic potential of rat osteosarcoma, while MMP2 could not be detected [28]. In stage IIB osteosarcoma, the immunohistochemical status of MMP9 has been shown to be strongly associated with overall and disease-free survival [29]. However, a recent meta-analysis including 18 studies covering 892 osteosarcoma patients demonstrated that although MMP9 might be a potential biomarker for osteosarcoma, a substantial heterogeneity exists among the different studies that could be attributed mainly to methodical issues [30]. In contrast to MMP9, several studies observed a more important role of MMP2 in osteosarcoma. Analysis of MMP expression and activity in osteosarcoma cell lines, biopsies and xenografts revealed a strong expression of MMP2 in culture supernatants from cell lines and in tumor xenografts while MMP9 expression was below the detection limit. MMP inhibitors significantly reduced the invasive potential of the analyzed cell lines indicating a crucial role of MMP2 in this process [31]. Further, analysis of osteosarcoma cells isolated from 23 pretreatment tumor tissues showed high MMP2 levels in all samples, while MMP9 could only be detected in one sample [32].

Based on these contradictory results and our own observations we assumed that, in contrast to total RNA or protein levels, the activity of these molecules in the tumor tissue might be a more valuable parameter with closer relation to their biological function. In fact, we could detect significant differences of MMP and TIMP activities between the analyzed good and poor responder groups that could not be observed for total RNA and protein levels. So far, the prognostic value of MMPs has been evaluated mainly concerning overall and event-free survival. Associations of MMPs with response to chemotherapy have not been studied yet. Current treatment protocols include a risk adapted adjuvant therapy according to the response to neoadjuvant chemotherapy that can only be assessed after tumor resection. Therefore, early stratification of osteosarcoma patients into low and high risk groups in order to improve outcome by means of a risk-adapted therapy is of high clinical relevance. However, until today, no biomarker could be established, allowing a risk stratification of osteosarcoma patients at time of diagnosis- neither regarding the response to current neoadjuvant chemotherapy protocols, nor to overall survival. Interestingly, we identified a switch from predominant MMP9 activity in the good responder group to predominant MMP2 activity in the poor responder group, suggesting that either loss of MMP9 activity or gain of MMP2 activity contributes to poor response to chemotherapy. As possible explanation for the different MMP activity patterns we identified diverse distributions of MMP inhibitors in the analyzed groups. We observed significant negative correlations of TIMP1 activity with MMP2 activity as well as TIMP2 and TIMP3 activity with MMP9 activity, suggesting that TIMP1 preferably inhibits MMP2 while TIMP2 and TIMP3 mainly act on MMP9, at least in the analyzed samples.

The observed association of MMP activity with response to chemotherapy was unexpected and the mechanisms involved are still unclear. Based on our previous results demonstrating a significant association of low tumor vascularization with good response to chemotherapy in osteosarcoma patients [33] and the fact that MMPs are crucially involved in the regulation of angiogenesis, we suppose that altered neoangiogenesis links up MMP activity with response to chemotherapy. MMP2 and MMP9 have been shown to be crucial for the “angiogenic switch” that occurs upon the initiation of tumor vascularization. A reduction in tumor growth and vascularization has been observed after transplantation of tumor cells into either MMP2 [34] or MMP9 [35] deficient mice, compared to wild-type mice. Further, expression of both, MMP2 and MMP9 was upregulated in angiogenic islets during carcinogenesis of pancreatic islets in transgenic mice. However, MMP knock-out mice revealed MMP9 as a key regulator of angiogenesis in this system, while MMP2 rather contributed to tumor growth [36]. In contrast, MMP2 was identified as the prominent matrix metalloproteinase in the angiogenic nodules in a rat chondrosarcoma model. Suppression of MMP2 activity by antisense oligonucleotides in the vascular nodules resulted in the loss of angiogenic potential both *in vitro* and *in vivo*, suggesting that MMP2 is required for the switch to the angiogenic phenotype [37]. These and further studies indicate that different MMPs are crucially involved in tumor angiogenesis that in turn influences response to chemotherapy.

To our knowledge, our study is the first showing a direct association of specific MMP activity patterns with response to chemotherapy in osteosarcoma, indicating that the ratio of MMP2 and MMP9 might be a valuable prognostic marker in pretreatment biopsies to predict the response to chemotherapy. We could further demonstrate that zymography is a powerful tool to quantify the activity of MMPs and TIMPs that might be closer related to their biological functions compared to total mRNA and protein levels. However, the small amount of samples available for our analyses has to be considered as limitation of our study. Quantification of MMP and TIMP activities required high quality cryo conserved tissue from osteosarcoma patients with known response to chemotherapy and adequate size and quality for RNA and protein extraction and further suitable for the production of cryosections. In contrast to formalin fixed paraffin embedded tissue such samples are much more limited. Larger studies are needed to validate our findings and to confirm the role of MMP2/MMP9 activity as prognostic marker and possible target for therapeutic interventions.

## Conclusions

So far, data on the prognostic value of MMPs and their inhibitors TIMPs in osteosarcoma are conflicting, most likely due to different technical approaches. Applying multiple zymography techniques we analyzed MMP and TIMP activities and identified a shift from MMP9 to predominant MMP2 activity in samples with poor response to chemotherapy that could not be observed for total mRNA or protein levels. Our preliminary data demonstrate that the ratio of MMP2/MMP9 activity might be a valuable prognostic marker for the response to chemotherapy that can easily be analyzed by zymography in tumor tissue samples. Our data further suggest that MMPs represent a possible target for therapeutic interventions.

### Ethics approval and consent to participate

This study has been approved by the Ethical Committee of the University of Heidelberg, Germany and written informed consent was obtained from all patients.

### Availability of data and materials

The datasets supporting the conclusions of this article are included within the main article.

## Abbreviations

bFGF: basic fibroblast growth factor
ECM: extracellular matrix
MMP: matrix metalloproteinase
TGF-ß: transforming growth factor beta
TIMP: tissue inhibitor of matrix metalloproteinases
VEGF: vascular endothelial growth factor
